# Is School Type Associated with Objectively Measured Physical Activity in 15-Year-Olds?

**DOI:** 10.3390/ijerph14111417

**Published:** 2017-11-20

**Authors:** Lovro Štefan, Maroje Sorić, Antonela Devrnja, Hrvoje Podnar, Marjeta Mišigoj-Duraković

**Affiliations:** Faculty of Kinesiology, University of Zagreb, 10 000 Zagreb, Croatia; maroje.soric@kif.hr (M.S.); antonela.devrnja@kif.hr (A.D.); hrvoje.podnar@kif.hr (H.P.); marjeta.misigoj-durakovic@kif.hr (M.M.-D.)

**Keywords:** children, adolescence, epidemiology, Sensewear Armband, high school type, energy expenditure

## Abstract

The main aims of this study were: (1) to determine the objectively assessed physical activity (PA) patterns in urban 15-year-old male and female adolescents according to school type and (2) to assess the differences in PA between school days and weekend days. In this cross-sectional study, participants were 187 secondary-school male and female adolescents (61.4% females) attending grammar and vocational schools. Patterns of PA were objectively evaluated using a multi-sensor body monitor for 5 consecutive days. Confounders assessed included biological age, socio-economic status, sum of 4 skinfolds, maximal temperature and the amount of rainfall. Males and females from grammar schools achieved higher total daily energy expenditure (TEE) and active energy expenditure (AEE) compared to their peers from vocational schools (TEE: 50 ± 12 kcal/kg/day vs. 47 ± 12 kcal/kg/day, *p* = 0.02; AEE: 23 ± 5 kcal/kg/day vs. vocational = 21 ± 6 kcal/kg/day, *p* = 0.04). No differences in time spent in light (LPA), moderate (MPA) or vigorous (VPA) physical activity were noted between the two groups (*p* = 0.16–0.43). Next, a significant decline in TEE and MPA between school days and weekends was observed (*p*< 0.001 and *p* = 0.02, respectively), while VPA remained the same throughout the week (*p* = 0.76). Weekly patterns of PA did not show differences by school type or gender (*p* for interactions = 0.21–0.50). In addition, significantly lower amount of MPA was accumulated during weekends compared to school days, resulting in lower TEE, regardless of school type or gender. Policies and strategies on PA in adolescents should focus vocational schools and weekend days.

## 1. Introduction

In the past two decades, lack of physical activity (PA) has become one of the major public health problems in the world [1]. A wealth of evidence has accumulated showing that regular PA reduces all-cause mortality and the incidence of cardiovascular diseases, type 2 diabetes and cancer, and enhances bone strength and psychological health [2].

The prevalence of insufficient PA in European school-going children is very high and ranges between 80 and 90% [1]. PA declines steadily during childhood, with the steepest drop being observed at the time of transition between primary and secondary school [3]. Consequently, the prevalence of physical inactivity is higher in secondary-school students compared to primary school students [4].

Correlates of PA are many [5], and recent studies have shown that the type of school can also influence adolescents’ PA [6,7,8,9,10]. It has been reported that students attending vocational schools are less physically active [6], spend more time in front of television and computer [7], and participate in sport less often [8,9] compared to their peers from grammar schools. Another potential factor influencing PA related to the type of school is socio-economic status (SES) [11]. Specifically, parents with lower educational level often have barriers to meeting the material costs of PA opportunities for their children [12]. Moreover, students attending grammar schools often report having higher SES, which potentially leads to higher participation in organized sports and lower prevalence of overweight [13]. Thus, it seems that both the type of school and SES contribute to PA levels in children and adolescents. Still, only a handful of studies have investigated the patterns of PA in children and adolescents according to school-type [6,7,8,9,10]. Furthermore, all these studies used subjective measures (questionnaires) to assess PA, which typically leads to overestimation of PA [14]. In order to create effective, school-oriented policies on PA, it is imperative to explore the impact of school type on children’s PA. In addition, to better understand the association of PA and school type, it is necessary to use objective methods of PA assessment that enable more accurate estimation of energy expenditure (EE).

Thus, the main aim of this study was to examine the associations of objectively assessed levels and weekly patterns of PA and the type of school in urban 15-year-old male and female adolescents.

## 2. Materials and Methods

### 2.1. Study Participants

This investigation is a part of the Croatian Physical Activity in Adolescence Longitudinal Study (CRO-PALS), an observational, longitudinal study designed to follow lifestyle habits of 15-year-old adolescents in the city of Zagreb (Croatia), during their secondary-school education. The sample size for the CRO-PALS study was estimated based on the assumption that primary analyses will comprise regression methods for longitudinal data. Sample size calculations performed with the Gpower [15] computer program suggested that a total sample of 311 individuals would be needed to detect small effects (*f*_2_ = 0.02) with 80% power and with alpha set at 0.05. However, since the CRO-PALS also aimed to generate prevalence estimates of insufficient physical activity and other risk factors for non-communicable disease, we had to increase the targeted sample to 900 individuals in order to achieve a 3% precision in the estimate of prevalence (assuming a total adolescent population in the city of Zagreb ≅ 40,000 and the projected prevalence of insufficient activity ≥70%). We relied on stratified two-stage random sampling procedures to select an adequately large representative sample of urban adolescents. First, all 86 secondary schools in Zagreb area were stratified by type: grammar schools/vocational schools/private schools. Next, at the first stage of random selection, based on the proportion of different types of schools and the average number of students per school of around 1500, 13 public (8 vocational and 5 grammar schools) and 1 private school (grammar school) were selected. During the second stage of randomization, half of the first grade classes in each of the selected schools were randomly selected. Finally, all 1408 students enrolled in the selected classes were approached and 903 agreed to participate (response rate = 64%).

The current investigation is based on a subsample of CRO-PALS participants in whom the level of PA was further examined by using a multiple sensor activity monitor. The main constraint on conducting objective PA assessment on all CRO-PALS participants was the number of available monitors for objective PA measurement [16]. Thus, from 14 schools initially enrolled in the study, 5 schools (3 vocational and 2 grammar) were randomly selected, which resulted in a total of 276 students participating in the objective PA assessment. To examine if the representativeness of the CRO-PALS sample has been preserved in the subsample selected for objective PA assessment, we compared 276 participants of this study to the rest of the CRO-PALS participants. These analysis indicated similar proportion of boys and girls (*p* = 0.43) and vocational school and grammar school attendees in both these groups (*p* = 0.23), as well as comparable values of biological age, BMI, physical fitness and SES (*p* = 0.37–0.52).

According to the inclusion criteria (described in the section “Physical activity assessment”), data from 187 adolescents were finally included in the analyses. To assess possible drop-out bias, we examined differences between participants with valid and non-valid data in terms of gender, age, BMI, SES, biological age, sum of 4 skinfolds (S4SF) and physical fitness (sit-ups/min and VO_2_max estimated by the 20-m shuttle-run test). No significant differences were observed between participants with valid and non-valid data in terms of gender (valid = B(39%)/G(61%) vs. non-valid = B(51%)/G(49%), Chi-square = 0.09, *p* = 0.09), age (valid = 15 ± 0.3 years vs. non-valid = 15 ± 0.4 years, *t* = 0.28, *p* = 0.78), BMI (valid = 21 ± 3 kg/m^2^ vs. non-valid = 22 ± 3 kg/m^2^, *t* = −1.90, *p* = 0.06), biological age (valid = 2 ± 0.7 vs. non-valid = 2 ± 0.8, *t* = 0.93, *p* = 0.35), S4SF (valid = 42 ± 15 mm vs. non-valid = 46 ± 19 mm, *t* = −1.86, *p* = 0.06), VO_2_max (valid = 41 ± 9 mLO_2_/kg/min vs. non-valid = 43 ± 10 mLO_2_/kg/min, *t* = −1.33, *p* = 0.18), sit-ups (valid = 21 ± 4x vs. non-valid = 21 ± 5x, *t* = −0.03, *p* = 0.97) and SES (valid: median = 2, IQR = 1–3 vs. non-valid: median = 3, IQR range = 2–4, Z = −0.58, *p* = 0.56).

### 2.2. Physical Activity Assessment

To objectively assess the level of PA, we used SenseWearArmband^TM^Pro3 (SWA) physical activity monitor (BodyMedia Inc., Pittsburgh, PA, USA). It relies on pattern recognition to estimate EE and the duration and intensity of PA. This device uses non-invasive sensors to measure different physiological parameters, such as skin temperature or body temperature. Together with height, weight, age, gender and handedness, the data obtained by the sensors are put in proprietary algorithms for estimation of EE and PA duration. The SWA has been previously found to be a valid tool for estimating EE and different levels of PA in children and adolescents [17].

The SWA device was placed on the right arm, above the m. triceps brachii, halfway between the olecranon and acromion processes. Before the main usage of the device, basic anthropological status (gender, height, weight, handedness and gender) was programmed into the SWA. Participants were instructed to wear the device for 5 consecutive days (3 schooldays and 2 weekend days) during the entire day and night, except during water activities or showering. For the analysis of the SWA data, the latest, child-specific algorithms were used (SenseWear Professional software v. 8.1; BodyMedia Inc., Pittsburgh, PA, USA). Participants were also given a physical activity diary to record activities during non-wear time. Consequently, duration of PA and EE expended during the period participants were not wearing the device were added to the SWA data based on the diary and according to the Compendium of PA for children and youth [18]. For the recording to be labeled valid, both of the following conditions had to be met: (1) a minimum of 10 h of awake time recorded per day and (2) a minimum of 3 valid days (including one weekend day) [19].

The intensity of PA was described through metabolic equivalents (METs). Time spent in activities requiring 4–7 METs was categorized as moderate physical activity (MPA), whereas activities requiring >7 METs were classified as vigorous physical activity (VPA). Light physical activity (LPA) was classified as time spent between 1.5 and 4 METs. Total energy expenditure (TEE) was divided by body weight of the participant and expressed as kilocalories/kilograms per day (kcal/kg/day). Active energy expenditure (AEE) represented energy expended in activities of at least light intensity and was also divided by body weight and expressed as kcal/kg/day. To determine the weekly average of TEE, AEE, LPA, MPA and VPA we multiplied the average school day value by 5 and the average weekend day value by 2 and then divided the score by 7, according to formula:

TEE, AEE, LPA, MPA, VPA = ((mean_schooldays_ × 5) + (mean_weekend days_ × 2))/7 (1)

### 2.3. Covariates

A list of covariates assessed includes BMI, subcutaneous body fat, biological age, socio-economic status and weather conditions.

Weight of the subjects was measured by using portable medical balanced scale to the nearest 0.1 kg. Subjects wore only shorts and T-shirts. Body height was taken by an anthropometer to the nearest 0.1 cm (GPM; Siber-Hegner & Co., Zurich, Switzerland). Body mass index (BMI) was calculated as body weight in kilograms divided by body height in meters squared (kg/m^2^).

Harpenden skinfold caliper(British Indicators, West Sussex, UK) was used to measure skinfold thickness to the nearest 0.2 mm on the right side of the body [20]. Skinfolds were measured at four sites as follows: (1) triceps- between the olecranon process and acromion process, (2) subscapular- below the tip of the scapula, taken with approximately 45° to the lateral side of the body, (3) suprailiac- above the iliac crest at the level of the anterior axillary line, (4) calf- at the maximal circumference, on the medial side. All skinfold measures were taken in triplicate and median values were used for analysis. The sum of 4 skinfolds (S4SF) was chosen as an indicator of body fat.

Biological age was estimated from the ratio of sitting height and height according to the formula and expressed as the number of years elapsed since peak height velocity [21].

SES was self-reported and assessed with the question: “What do you think your socioeconomic status is, compared to other peers?” The responses were arranged along a Likert-type, five-point scale: (1) extremely above average, (2) above average, (3) average, (4) below average and (5) extremely below average.

Finally, data on the maximal daily temperature (T_max_) and the amount of rainfall (mm of rainfall) during the days the SWA device was worn were obtained from the Croatian National Meteorological and Hydrological Service [22].

### 2.4. Data Analysis

Before the main analysis, we checked all variables for normal distribution by using Kolmogorov-Smirnov test. If certain variable was not normally distributed, logarithmic transformation was applied. Data are presented as mean (standard deviation) for normally, or as median (inter-quartile range) for non-normally distributed data. Differences in physical characteristics of adolescents from vocational and grammar schools were determined using analysis of variance (ANOVA) and Kruskal-Wallis test for numerical, and Chi-square test for categorical variables. The main effect of gender and school type in PA measures, as well as the interaction effect between gender and type of school, were analyzed by 2-way analyses of covariance (ANCOVA) adjusted for biological age, S4SF, SES, T_max_ and mm of rainfall. Next, differences in PA during the week (Monday–Friday) and on weekend days (Saturday–Sunday) were examined using repeated measures ANCOVA with gender and the type of school as between-subject factors and adjusted for the same covariates as above. Two-sided *p*-values were calculated and significance was set at α < 0.05. All the analyses were performed using Statistical Packages for Social Sciences v.23 (SPSS, Chicago, IL, USA).

## 3. Results

Before performing main analyses, we wanted to explore possible effect of monitoring duration on the average level of participants’ PA. Of 187 valid participants, 109 participants (58 %) completed the full 5 days of recording, 51 participants (27%) had 4 valid days of recording, while 27 (15%) of them had 3 days of recording. As no significant differences in any of the PA measures were found between groups of participants wearing the SWA device 3, 4 or 5 days (*p* = 0.301–0.935), all participants were pooled for further analysis. Next, very similar wearing time was recorded in adolescents from both types of school (grammar = 16.6 ± 3 h/day vs. vocational = 16.1 ± 3 h/day, *t* = 1.03, *p* = 0.30) and in both genders (boys = 16.6 ± 3 h/day vs. girls = 16.2 ± 3 h/day, *t* = 0.87, *p* = 0.38).

Basic characteristics of the study participants are presented in Table 1. Boys and girls attending vocational schools reported having slightly lower SES compared to their peers from grammars schools (*p* = 0.01). There were no significant differences in other characteristics assessed (*p* > 0.05).

Table 2 shows PA levels according to gender and school type (grammar vs. vocational). Boys had higher TEE and AEE and spent more time in both MPA and VPA than girls (*p* ≤ 0.001–0.04). Next, boys and girls attending grammar schools exhibited higher values of TEE [50.3 (12.4) kcal/kg/day] compared to their peers vocational (TEE = 47.0 (12.0) kcal/kg/day) schools (*p* = 0.02). Moreover, adolescents from grammar schools had higher AEE compared with vocational students (*p* = 0.04). In contrast, no differences in PA duration of any intensity between male and female adolescents attending different types of school were found (*p* = 0.16–0.43). Finally, no significant interactions between gender and the type of school were found (*p* = 0.21–0.96).

**Figure 1:**
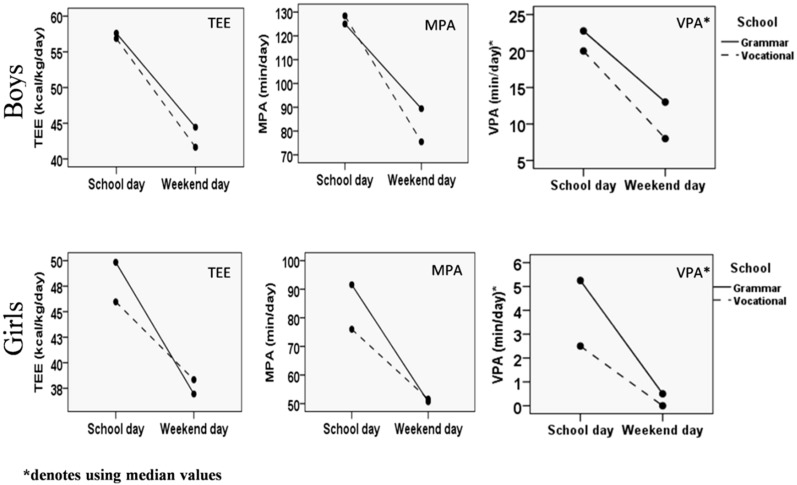
Changes in total energy expenditure (TEE), moderate physical activity (MPA) and vigorous physical activity (VPA) between school days and weekends in boys and girls attending grammar or vocational schools.

Figure 1 shows the differences in TEE, MPA and VPA between school days and weekend days stratified by gender and the type of school. The highest TEE values were recorded in grammar school boys during school days (57.9 ± 16.5 kcal/kg/day) and the lowest in grammar school girls on weekends (36.9 ± 8.2 kcal/kg/day). When both boys and girls were observed as a group, the main effect for time showed significant decrease in TEE from school day to weekend day (51.8 ± 15.3 kcal/kg/day vs. 39.3 ± 9.0 kcal/kg/day, *F* = 12.66, *df* = 1, *p* < 0.001, η^2^ = 0.07). Non-significant time x gender x school type interaction suggests comparable magnitude of the decline in TEE in both genders and types of school (*F* = 1.40, *df* = 3, *p* = 0.25, η^2^ = 0.03). Similarly, the duration of MPA was almost twice higher during the week (102 ± 58 min/day) compared to weekends (62 ± 50 min/day, *F* = 5.19, *df* = 1, *p* = 0.02, η^2^ = 0.03). Specifically, boys from vocational schools accumulated the highest amount of MPA during school days (126 ± 48 min/day), while the lowest amount was recorded in girls from vocational schools on weekends (52 ± 46 min/day). Boys from vocational schools and girls from grammar schools accumulated approximately 45% less MPA on weekend day than during school day, while the corresponding difference in grammar school boys and vocational school girls amounted to approximately 30%. However, time x gender x school type interaction failed to reach statistical significance (*F* = 1.63, *df* = 3, *p* = 0.18, η^2^ = 0.03). Finally, no significant time effect was observed between school days and weekends regarding VPA (8 (2–22) min/day vs. 2 (0–13) min/day, *F* = 1.08, *df* = 1, *p* = 0.30, η^2^ = 0.01). However, the highest VPA values were observed in grammar school boys during school day (VPA = 22 (9–124) min/day) and the lowest in vocational school girls on weekends (VPA = 2 (0–23) min/day). Again, non-significant time x gender x school type interaction indicated similar patterns of change in VPA in all 4 groups (*F* = 0.05, *df* = 3, *p* = 0.83, η^2^ < 0.001).

## 4. Discussion

This study investigated patterns of objectively assessed PA among 15-year-old male and female adolescents attending grammar and vocational schools. The main finding of this investigation was that boys and girls from grammar schools exhibited higher TEE and AEE compared with their peers from vocational schools, although no significant differences regarding the duration of daily LPA, MPA and VPA between these two groups of adolescents were found.

Similar to the present study, previous studies using subjective methods to assess PA have shown that compared with the vocational school program, boys and girls from grammar schools are more physically active [6], and participate in sports more often [8,9]. Although we noted higher AEE in grammar school adolescents compared to their peers from vocational schools, PA duration was not statistically different. However, due to a rather small sample size (*N* = 187), we have to acknowledge that the study may have been underpowered in some aspects. Specifically, with the power of 0.8, number of participants of 187 and alpha < 0.05, we could detect the effect size of 0.25, which is considered to be a medium-sized effect. Since variability in MPA, and especially VPA in study participants was quite high, only differences greater than 12 min/day between the two groups of adolescents could have been detected in our study. Despite observing non-significant school effect for the duration of LPA, MPA and VPA, we noted that the type of school had a significant effect on AEE, that is, adolescents from grammar schools expended more energy in physical activity compared with adolescents form vocational schools. AEE was defined as energy expenditure during non-sedentary time, which includes LPA, MPA and VPA combined. Thus, although the duration of PA was similar in adolescents from both school types, mean intensity of PA was obviously higher in adolescents attending grammar school. Next, as it was shown in some previous studies that students from vocational schools are less engaged in organized sports [8,9] this could also be a potential reason for the higher average intensity of daily activities noted in grammar school students in our study. In this study, however, participation in out-of-school organized sports seems to be similar for both school types (data not shown), which is inconsistent with previous findings in Australian adolescents [8,9]. Such divergent findings possibly result from different social contexts, geographical settings, time of the year when the study was conducted and different methodology used in the aforementioned studies. Finally, despite similar PA duration being noted in both groups, lower TEE observed in male and female adolescents from vocational school peers compared to their peers from grammar schools could possibly translate to higher risk of weight gain during secondary school [23]. Indeed, larger prevalence of obesity in vocational compared to academic programs has been previously reported [9].

The average amount of PA in our study was significantly higher than the recommended 60 min of MVPA daily. Specifically, boys engaged in 142 min of MVPA on average, while girls averaged roughly 83 min of MVPA per day (data not shown). However, most of this PA was of moderate intensity, especially in girls. Our results can be compared with the PA patterns of 15-year-old students from 4 European countries [24]. In this study, 15-year-old boys engaged in approximately 100 min of MVPA, while girls accumulated around 70 min, which is somewhat lower than noted in this study. The reasons for this difference are multiple. First, it has been reported that the underestimation of EE during non-weight-bearing activities and activities involving upper body is much smaller for the SWA device used in this study compared to accelerometers used by Riddoch et al. [24]. Second, PA behavior tends to be differed across the year cycle, with the highest PA level during spring and the lowest during winter [25]. As highlighted before, our study was conducted between March and June, while the study by Riddoch et al. [24] was spread throughout the whole year [24]. Third, different geographical, social and environmental context could also be driving this difference [16].

In the present study, boys showed higher TEE, AEE, MPA and VPA compared to girls. This is consistent with numerous previous epidemiological studies that have used the objective methods to assess PA in similar age groups [24,26,27], although the difference between genders is not identical. Specifically, De Baere et al. [27] showed that boys spent approximately 15 min/day and 9 min/day longer in moderate and vigorous PA, compared with girls. Another study by Riddoch et al. [24] showed that boys engaged in 99 min/day and girls in 73 min/day of MVPA (−26%), which is lower compared with the mean difference in MVPA from our study (−42%) [24,27]. These differences partly stem from non-identical age groups and different PA assessment methods across the studies. However, the observed trend for adolescent boys engaging in more PA than girls is clear.

Finally, a significant decline in PA during weekend days compared to school days in both boys and girls and schools was observed. However, our results showed non-significant main effect for the type of school, indicating that male and female adolescents from both grammar and vocational schools had similar magnitude of change through the week. To the best of our knowledge no prior studies have explored the changes in PA between the school day and weekend day, according to school type. Studies that tracked PA behavior through the week have shown mixed results, with some reporting higher accumulation of PA during school days [16,28,29], while others reported higher amount of PA during weekend days [19,30].

This study has several strengths. First, we used a multi-sensor PA monitor to objectively evaluate PA patterns. Second, we adjusted for numerous covariates (biological age, SES, S4SF, T_max_ and the amount of rainfall). Biological age was included rather than chronological, since it has proven to be a better predictor of participation in PA [31]. Lastly, to account for seasonal variation of PA, not only that we confined PA assessment to 3 spring months, but we managed to account for weather conditions by adjusting for T_max_ and the amount of rainfall in the data analyses.

However, this study also has several limitations. First, although 5-day assessment period used in this study has been previously shown to yield reliable estimates of PA, by increasing the assessment period to the whole week more accurate estimates of PA patterns would be generated [32]. Second, SES is a very complex concept which has been shown to influence PA levels of adolescents. In this study, as a proxy of SES, we used a one-item question, thus ignoring the complex nature of SES. Although we adjusted for it, we did not include more detailed information about the SES of students, such as parents’ education, monthly income etc. Therefore, some residual confounding of the relationship between PA and school type by SES is possible. Third, the differences in PA cannot fully be attributed to school environment, since Sensewear Armband cannot analyze the domains of PA or the type of activity participants engaged in, but only intensity and duration. Thus, it is possible that out of school activities contributed the differences between the male and female adolescents from different schools. Future studies should provide more detailed information about the SES of the participants. Finally, we randomly selected schools and classes for the purpose of this study and achieved an acceptable response rate. Nevertheless, more physically active families are more prone to participating in the studies of such nature. Thus, potential selection bias cannot be excluded.

## 5. Conclusions

The average amount of PA observed in our study was rather high, although in girls very little VPA was recorded. Male and female adolescents attending grammar schools showed higher TEE and AEE compared to their peers from vocational schools, although the duration of PA was similar in children from both school types. In addition, we noted a significant decrease in PA on weekends compared to school days, regardless of the type of school or gender. Policies and strategies aimed at increasing PA should focus vocational schools. Moreover, interventions should be extended beyond the school-week to cover weekends also. Future studies using objective methods of PA assessment should include longer periods of monitoring and larger sample size in order to examine PA patterns across school-types more accurately.

## Figures and Tables

**Table 1:** Basic characteristics of the study participants, stratified by the gender and the type of school.

Study Variables	Boys (*N* = 72)		Girls (*N* = 115)		*p*-Value
	Grammar (*N* = 41)	Vocational (*N* = 31)	Grammar (*N* = 64)	Vocational (*N* = 51)	
Age (years)	15.4 (0.4)	15.7 (0.4)	15.6 (0.3)	15.6 (0.4)	0.18
Height (cm)	178 (8)	176 (6)	167 (5)	165 (5)	0.09
Weight (kg)	66 (11)	67 (12)	59 (9)	57 (9)	0.08
BMI (kg/m^2^)	21 (3)	21 (9)	21 (2)	20 (2)	0.06
Biological age (years from Peak Height Velocity)	1.8 (0.7)	2.0 (0.6)	2.9 (0.4)	2.8 (0.4)	0.93
S4SF (mm)	33 (15)	37 (16)	49 (16)	44 (13)	0.8
SES * (scale)	2 (2–3)	3 (2–4)	2 (2–4)	3 (2–4)	0.01

* median (lower quartile-upper quartile); *p*-values for the main effect of the type of school are presented.

**Table 2:** Physical activity measures stratified by gender and the type of school.

Study Variables	Boys (*N* = 72)		Girls (*N* = 115)		*p*-Values *		
	Grammar	Vocational	Grammar	Vocational	Gender	School	Gender * School
	*N* = 41	*N* = 31	*N* = 64	*N* = 51			
	Mean (SD)	Mean (SD)	Mean (SD)	Mean (SD)			
TEE (kcal/kg/day)	55.1 (12.6)	52.4 (10.2)	47.7 (11.5)	42.6 (9.8)	0.04	0.02	0.21
AEE (kcal/kg/day)	24.3 (5.2)	22.6 (6.5)	21.8 (5.6)	20.0 (6.9)	<0.01	0.04	0.96
LPA (min/day)	321 (89)	318 (123)	351 (111)	316 (128)	0.3	0.43	0.36
MPA (min/day)	114 (54)	111 (42)	81 (43)	70 (50)	<0.001	0.32	0.39
VPA (min/day) #	24 (9-47)	21 (7–39)	4 (1–12)	2 (0–8)	<0.001	0.09	0.42

# median (lower quartile-upper quartile); * *p*-values from ANCOVA of the main effects of school and gender and for gender * school interaction adjusted for biological age, S4SF, SES, T_max_ and the amount of rainfall.

## Abbreviations

AEE	active energy expenditure
ANCOVA	analysis of covariance
BMI	body-mass index
CRO-PALS	Croatian Physical Activity in Adolescence Longitudinal Study
EE	energy expenditure
LPA	light physical activity
MET	metabolic equivalent
MPA	moderate physical activity
PA	physical activity
SES	socioeconomic status
S4SF	sum of four skinfolds
TEE	total energy expenditure
VO_2max_	maximal oxygen uptake
